# AVATAR therapy for auditory verbal hallucinations in people with psychosis: a single-blind, randomised controlled trial

**DOI:** 10.1016/S2215-0366(17)30427-3

**Published:** 2018-01

**Authors:** Tom KJ Craig, Mar Rus-Calafell, Thomas Ward, Julian P Leff, Mark Huckvale, Elizabeth Howarth, Richard Emsley, Philippa A Garety

**Affiliations:** a Department of Health Service and Population Research, Institute of Psychiatry, Psychology and Neuroscience, King's College London, London, UK; b Department of Psychology, Institute of Psychiatry, Psychology and Neuroscience, King's College London, London, UK; c Department of Mental Health Sciences, Royal Free and University College Medical School, London, UK; d Department of Speech, Hearing and Phonetic Sciences, University College London, London, UK; e Centre for Biostatistics, School of Health Sciences, The University of Manchester, Manchester Academic Health Science Centre, Manchester, UK

## Abstract

**Background:**

A quarter of people with psychotic conditions experience persistent auditory verbal hallucinations, despite treatment. AVATAR therapy (invented by Julian Leff in 2008) is a new approach in which people who hear voices have a dialogue with a digital representation (avatar) of their presumed persecutor, voiced by the therapist so that the avatar responds by becoming less hostile and concedes power over the course of therapy. We aimed to investigate the effect of AVATAR therapy on auditory verbal hallucinations, compared with a supportive counselling control condition.

**Methods:**

We did this single-blind, randomised controlled trial at a single clinical location (South London and Maudsley NHS Trust). Participants were aged 18 to 65 years, had a clinical diagnosis of a schizophrenia spectrum (ICD10 F20–29) or affective disorder (F30–39 with psychotic symptoms), and had enduring auditory verbal hallucinations during the previous 12 months, despite continued treatment. Participants were randomly assigned (1:1) to receive AVATAR therapy or supportive counselling with randomised permuted blocks (block size randomly varying between two and six). Assessments were done at baseline, 12 weeks, and 24 weeks, by research assessors who were masked to therapy allocation. The primary outcome was reduction in auditory verbal hallucinations at 12 weeks, measured by total score on the Psychotic Symptoms Rating Scales Auditory Hallucinations (PSYRATS–AH). Analysis was by intention-to-treat with linear mixed models. The trial was prospectively registered with the ISRCTN registry, number 65314790.

**Findings:**

Between Nov 1, 2013, and Jan 28, 2016, 394 people were referred to the study, of whom 369 were assessed for eligibility. Of these people, 150 were eligible and were randomly assigned to receive either AVATAR therapy (n=75) or supportive counselling (n=75). 124 (83%) met the primary outcome. The reduction in PSYRATS–AH total score at 12 weeks was significantly greater for AVATAR therapy than for supportive counselling (mean difference −3·82 [SE 1·47], 95% CI −6·70 to −0·94; p<0·0093). There was no evidence of any adverse events attributable to either therapy.

**Interpretation:**

To our knowledge, this is the first powered, randomised controlled trial of AVATAR therapy. This brief, targeted therapy was more effective after 12 weeks of treatment than was supportive counselling in reducing the severity of persistent auditory verbal hallucinations, with a large effect size. Future multi-centre studies are needed to establish the effectiveness of AVATAR therapy and, if proven effective, we think it should become an option in the psychological treatment of auditory verbal hallucinations.

**Funding:**

Wellcome Trust.

## Introduction

Auditory verbal hallucinations, which are typically of a derogatory and threatening nature, are reported by approximately 60–70% of people with schizophrenia.^3^ Although pharmacological therapy is effective at reducing hallucinations in many people, approximately 25% of people with psychotic conditions continue to experience them.^4^ Cognitive behavioural therapy for psychosis is also helpful for many people, although average effect sizes are in the small to moderate range,^2^ and training and resource requirements mean that, in practice, therapy is delivered to only a fraction of those who might benefit.^5^ Consequently, there is considerable interest in the development of novel therapies that draw on the principles of cognitive behavioural therapy for psychosis but which are shorter, specifically targeted at auditory verbal hallucinations, and are capable of being delivered by a wider workforce.

Several novel therapies build on the perspective that auditory verbal hallucinations are experienced as coming from entities that have personal identities, speak with purpose, and with whom the hearer establishes a personal relationship. The operation of power within this relationship is viewed as crucial.^6^, ^7^ The voice is typically experienced as dominant (even omnipotent), with the voice-hearer assuming a submissive role characterised by feelings of inferiority and powerlessness that can reflect social relationships more generally.^8^ In light of this finding, explicitly relational and interpersonal approaches have been developed that locate voices (and voice relationships) within the person's biographical context^9^ and target key interpersonal dimensions such as power and proximity.^1^, ^7^

AVATAR therapy belongs to this new wave of relational approaches but, uniquely, the voice-hearer's experiences are brought into therapy in a new way, allowing a face-to-face interaction with a digital representation (avatar) whose speech closely matches the pitch and tone of the persecutory voice. The therapist (switching between speaking as therapist and as avatar) facilitates a dialogue in which the voice-hearer gradually gains increased power and control within the relationship, with the initially omnipotent voice loosening its grip over the hearer by becoming more conciliatory over time.

**Research in context**

**Evidence before this study**

The previous pilot study that prompted this randomised controlled trial was unique in the use of specific digital technology to enable a trialogue between therapist, patient, and a simulation of the auditory hallucination experienced by the patient. We searched Medline, PubMed, and PsychInfo databases for articles published in English between Jan 1, 1950, and June 30, 2017, with search terms “auditory hallucinations”, “voices”, “psychosis”, “schizophrenia”, AND “psychological therapy”, “CBT”, and “voice dialogue”. We also reviewed published meta-analyses of cognitive behaviour therapy for hallucinations. As we expected, there is substantial literature in which assessment of auditory hallucinations have been reported as one component of a wider therapy, typically with small to moderate effect sizes. In addition to the pilot study of AVATAR therapy, there were two other specifically relational approaches to distressing voices: voice dialogue, which to date has been reported as single cases, an ongoing case series, and a pilot controlled trial; and Relating Therapy for voices, which has shown a reduction of voice-related distress compared with treatment as usual. Neither use digital representations of patient experiences in therapy.

**Added value of this study**

We corroborated the results of the earlier pilot study in a larger, powered, randomised controlled trial comparing AVATAR therapy with an augmented supportive counselling control condition. The effect size on our primary outcome is greater than that reported by meta-analyses of cognitive behaviour therapy for psychosis.

**Implications of all the available evidence**

Traditional cognitive behaviour therapy for psychosis is a lengthy therapy that is delivered by highly trained therapists and is, consequently, a scarce resource, which achieves small to moderate effects on auditory verbal hallucinations. Results from our study suggest a benefit for briefer therapies that employ digital representations of voices in dialogue and are focused on specific target processes, which could be incorporated within a broader therapy (such as cognitive behaviour therapy for psychosis) or offered as a standalone approach.

A pilot study^10^ that compared AVATAR therapy with a treatment as usual in 26 patients who had a longstanding single or dominant persecutory voice found significant reductions in the frequency, distress, omnipotence, and malevolence of the voice. We report the results of a larger, randomised controlled trial^11^ that compared AVATAR therapy with an augmented supportive counselling intervention. There were three objectives: to test the clinical efficacy of AVATAR therapy compared with supportive counselling, to explore explanatory mechanisms of action and moderators for AVATAR therapy, and to determine preliminary estimates of cost-effectiveness of AVATAR therapy.

This paper addresses the primary objective—to test clinical efficacy—with the following hypotheses: AVATAR therapy will be more effective in reducing the frequency and severity of auditory verbal hallucinations, by comparison with supportive counselling, at 12 weeks; AVATAR therapy will be more effective in reducing the reported omnipotence and malevolence of auditory verbal hallucinations, by comparison with supportive counselling, at 12 weeks; and the improvements attributable to AVATAR therapy will be maintained at 24 weeks follow-up. The other two objectives will be addressed in subsequent publications.

## Methods

### Study design and participants

The study was a single-blind, randomised controlled trial done in the South London and Maudsley NHS Trust. Potential participants were referred to the study by their treating clinician in routine clinical service. All referrals were screened for eligibility against the following inclusion criteria: having had distressing auditory verbal hallucinations for at least 12 months in the context of a diagnosis of a schizophrenia spectrum disorder (ICD-10 F20–29) or affective disorder with psychotic symptoms (ICD-10 F30–39 subcategories with psychotic symptoms), currently a patient of NHS psychiatric services, older than 18 years, and able to speak and read English. All participants had been taking antipsychotic medication before the trial but their auditory verbal hallucinations had been unresponsive or only partially responsive to previous treatment. Participants were excluded if they were unable to give informed written consent; were currently receiving psychological therapy for psychosis, including attending so-called hearing voices groups; were refusing medication; had a diagnosis of organic brain disease, learning disability, or primary substance dependency; and heard voices in a language not spoken by the therapists. All participants gave written informed consent.

The study was approved by the London-Hampstead Research Ethics Committee (reference 13/Lo/0482). The trial protocol has been published elswhere.^11^ The study was overseen by an independent trial steering committee and a separate Data Monitoring and Ethics Committee.

### Randomisation and masking

All participants continued to receive standard psychiatric care, with the agreement that existing medication was to remain unchanged over the duration of the trial unless otherwise determined by clinical need. Participants were randomly assigned (1:1) to receive AVATAR therapy or supportive counselling with randomised permuted blocks (block size randomly varying between two and six). Randomisation was done on completion of baseline assessments through an independent web-based service provided by the UKCRC Registered Clinical Trials Unit at King's College London (registration number 053) to maintain allocation concealment. Participants were informed of their allocation by a therapist.

All assessments were done by research assessors who were masked to therapy allocation. To avoid unmasking, we ensured that assessors did not have access to clinical records after the baseline (pre-randomisation) assessment or access to the therapy database at any stage, that all assessments were done at sites remote from the clinic, and that participants were reminded before each assessment not to disclose their allocation.

### Procedures

Therapy in both groups was provided at a single clinical location. AVATAR therapy was delivered by experienced clinicians skilled in psychological therapies. Participants first created a computerised representation of the entity that they believed was the source of their main voice. After completing the set-up of the avatar in an introductory session, which included a comprehensive assessment of the voice(s) and included verbatim content, the therapy was delivered over six weekly 50-min sessions. 10–15 min of each session involved face-to-face work with the avatar, wherein the therapist facilitated a direct dialogue between the participant and the avatar. Participants sat in one room facing their avatar on a computer monitor. The therapist sat in a second room with a control panel that allowed them to speak in his or her own voice, or as the avatar. A video link allowed the therapist to see and hear the participant's responses, enabling them to adjust therapeutic interventions and modify the avatar interaction according to the unfolding dialogue. The progress of sessions was determined by a discussion in each session concerning any change in severity, malevolence, or frequency of the voices. All sessions were audio recorded and a copy of the avatar dialogue was provided on an MP3 player to the participant with instructions to listen to the recording at home, especially when they heard the voice(s). The content of the sessions has been described elsewhere.^12^ Briefly, therapy proceeds through two phases. Phase one (typically sessions one to three) involved exposure to the avatar speaking the typical verbatim content of the participant's voices while the therapist encouraged assertive responding—eg, that the person tell the avatar that they are no longer prepared to accept these threats and insults and to challenge any apparent misconceptions the avatar seems to have. In phase two (typically sessions four to six), the dialogue gradually evolved as the avatar conceded ground and acknowledged the strengths and good qualities of the participant. There is an explicit focus on self-esteem and acknowledgments of the participant's strengths and capabilities.

Therapists used a detailed therapy manual written for the trial by the team, developed from an earlier brief guide provided by JPL, who also provided initial training and consultation through periodic attendance at weekly group supervision meetings. The complete audiotaped clinical record of 64 sessions across a random selection of 12 participants (including discussions before and after active dialogue) was rated by JPL against a 25-item scale developed to assess adherence to the manualised approach and skill in delivery.

The control condition, supportive counselling, was delivered by graduate assistant psychologists who were recruited on the basis of extensive experience of working therapeutically in a psychosis context. They were trained and closely supervised throughout by the therapy co-ordinator (TW). The intervention comprised a manual-based, face-to-face supportive counselling approach adapted with permission from that employed by the SoCRATES Trial Group.^13^ Whereas the inherited manual proposed activities such as board games and listening to music together, we wanted to provide more than a simple attention or time control and augmented the manual to deliver an emotion-focused psychological intervention that facilitated exploration of issues of fundamental importance in the person's life, in the context of an empathic, non-directive therapeutic relationship.^14^ At the same time, we wanted to avoid the interpersonal treatment targets of AVATAR therapy (ie, shift in power or control in the relationship with the voice[s])

Typical themes of counselling included improving quality of life, issues of identity or belonging, coming to terms with past trauma, and identifying personal resources and qualities. Counselling was delivered over the same number and duration of sessions as AVATAR therapy. At the end of each session, participants recorded a weekly positive message onto an MP3 player to listen back to during the week (matching the use of MP3 recordings in the AVATAR therapy group).

An independent counselling psychologist rated 67 sessions across 14 participants for fidelity to the manual and evidence of key therapeutic processes, using core items of the counselling adherence scale.^15^ For the counselling adherence scale, there are seven items, scored 0 (no evidence) to 4 (very good evidence).

At the end of therapy, participants in both groups were given a therapy summary letter and a copy of this was sent to their responsible clinician in routine care. No attempt was made to intervene or monitor participants between the end of therapy and the assessment points.

Discontinuation of therapy from either group was defined by one or more of the following criteria: non-attendance at three consecutive sessions, the participant decided to discontinue, or discontinuation recommended by the therapist or peer supervision group. Following the protocol, therapy could also be terminated if the participant reported complete absence of any voices for at least three consecutive sessions. The total number of sessions could also be extended by up to three sessions when there was a rationale for probable benefit, agreed by therapy team consensus.

Audiotaped assessments were carried out by trained research staff at baseline, 12 weeks, and 24 weeks after randomisation. We assessed inter-rater reliability using intra-class correlations, two-way mixed-effects model, and absolute agreement type for the main outcome measure in 25 (20%) of 125 interviews. We calculated indices for each of the items and total score of this scale.

### Outcomes

The prespecified primary outcome was the total score (0–44) on the Psychotic Symptom Rating Scales, auditory hallucinations subscale (PSYRATS–AH^16^) at 12 weeks.

The secondary outcome measures of voices were dimensional subscales of the PSYRATS-AH:^17^ voice frequency (frequency, duration, and disruption items) and voice distress (negative content, distress, and control items), Revised Beliefs about Voices Questionnaire^18^ (BAVQ-R; perceived malevolence, omnipotence, and benevolence subscales), the Voice Acceptance and Action Scale^19^ (VAAS; acceptance and action subscales), Voice Power Differential Scale^20^ (VPDS; power and assertiveness subscales).

The other secondary outcomes measures were scores for the Scale for Assessment of Positive and Negative Symptoms^21^ (SAPS and SANS), Psychotic Symptoms Rating Scale–Delusions^16^ (PSYRATS-DEL), Depression Anxiety and Stress Scale^22^ (DASS-21), Calgary Depression Scale,^23^ Rosenberg self-esteem,^24^ Manchester Short Assessment of Quality of Life (MANSA),^25^ and the Maudsley Addiction Profile (MAP).^26^

Information about possible adverse events was monitored for the duration of the study, up to week 24 of follow-up. Possible adverse events included hospital admissions (due to physical or mental health deterioration), crisis team involvement, self-harming behaviour and suicide attempts, and violent incidents necessitating police involvement (whether the participant was victim or perpetrator). All adverse events were reported to the trial steering, data monitoring and ethics, and research ethics committees.

We paid close attention to the occurrence of any adverse events that might be attributed to either therapy, including whether the AVATAR therapy sessions were followed by any therapy-specific issues such as seeing the avatar outside of the sessions. Therapy was to be terminated in the event of any evidence of therapy being associated with significant increased distress, risk of harm to self or others, or both.

### Statistical analysis

We planned to enrol 142 participants. In the pilot study^10^ there was a clinically meaningful five-point greater change in the total PSYRATS–AH score, favouring the AVATAR condition with an effect size (Cohen's *d*) of approximately 0·8. Supportive therapy typically achieves a modest effect of *d*=0·2. On the assumption that a replication study might be expected to achieve broadly similar results, we calculated that a sample size of 71 in each group would have 90% power to detect a net effect size of 0·6, using a two-group *t*-test with a 0·05 two-sided significance level, while also allowing for a 20% loss to follow-up.

We did the statistical analysis with Stata (version 14.1). We report on all outcomes related to our primary (efficacy) objective, as specified in our published protocol and statistical analysis plan, and as agreed with the data monitoring and ethics committee before any analysis was done. All analyses used the original randomised groups, including participants with observed outcome data. Descriptive statistics within each randomised group are presented for baseline values, including counts and percentages for binary and categorical variables and means and SDs for continuous variables and counts of missing values. No statistical tests were done on baseline measures.

We analysed the primary outcome (PSYRATS–AH total score at 12 weeks) using a linear mixed-effects model allowing for the baseline measurement of PSYRATS–AH and randomisation as fixed effects. Because of the nested trial design, we used mixed models to include a random intercept for each therapist in the two randomised groups, allowing for differential clustering by group. We analysed secondary outcome measures using the same modelling approach. We also analysed the primary and secondary outcomes at 24 weeks using mixed models. We examined if baseline factors were associated with missing outcomes using logistic regression, and no significant predictors were present once baseline scores and randomised group were accounted for.

In the event that a participant reported a complete absence of auditory verbal hallucinations for the entire week at either 12 or 24 weeks, the best possible score was imputed for BAVQ-R, VAAS, and VPDS scales, which cannot be used in the absence of auditory verbal hallucinations. Missing data on other measures were pro-rated if more than 90% of the total items were completed, otherwise the measure was considered as missing. We used logistic regression to examine if baseline factors were associated with missing outcomes, and no significant predictors were present once baseline scores and randomised group were accounted for.

The trial was prospectively registered with the ISRCTN registry, number 65314790.

### Role of the funding source

The funder of the study had no role in study design, data, collection, data analysis, data interpretation, or writing of the report. The funder reviewed and approved the application for the trial. RE had full access to all the data in the study and the corresponding author had final responsibility for the decision to submit for publication.

## Results

**Figure 1:**
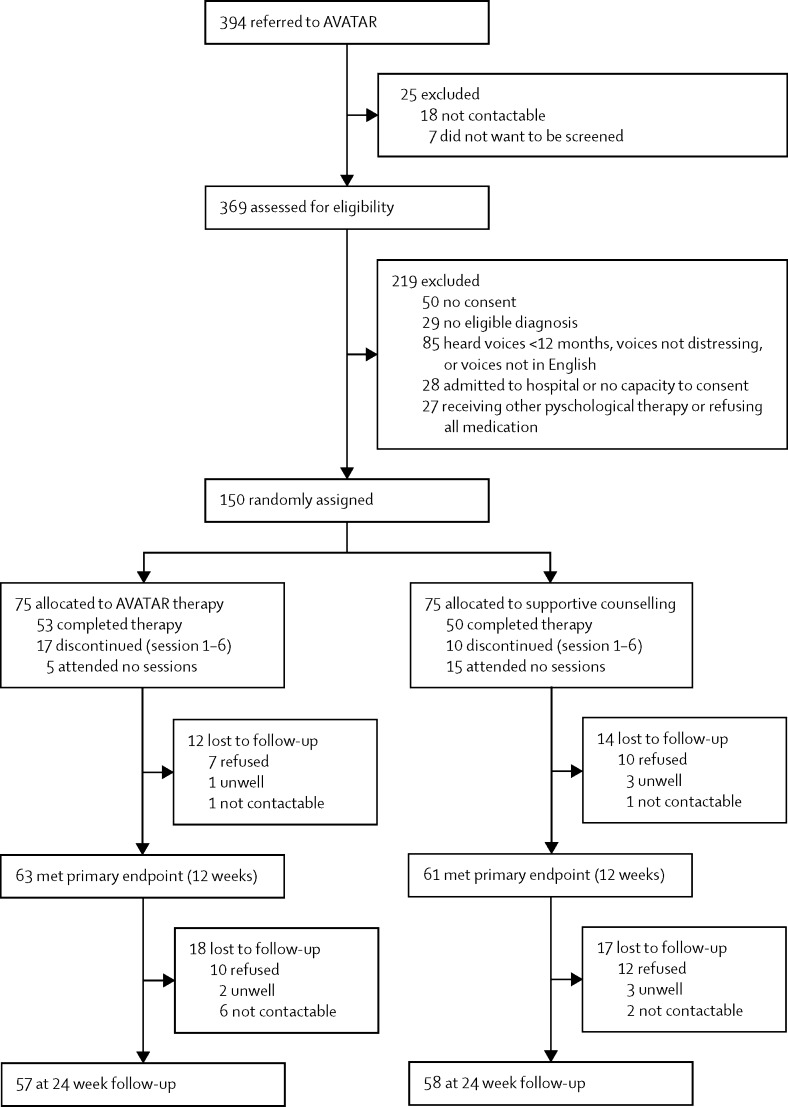
Trial profile Numbers lost to follow-up are cumulative in relation to the total allocated at the start of the study.

Between Nov 1, 2013, and Jan 28, 2016, there were 394 referrals to the study (figure 1). 25 were excluded before eligibility screening because they could not be contacted or refused to meet the researcher for screening. We assessed 369 participants, of whom 150 were eligible and gave informed consent. Participants were randomly assigned to AVATAR therapy (n=75) or supportive counselling (n=75; figure 1). Reasons for participant exclusion at eligibility assessment were: not providing consent (n=50); not meeting diagnostic criteria (n=29); not hearing distressing voices, hearing voices for less than 12 months, or reporting voices not speaking in English (n=85); being acutely unwell in hospital, not having the capacity to give consent, or both (n=28); and several other reasons (eg, severe physical ill health, pregnancy, and refusing to take medication; n=27). The majority of participants (n=369) were referred by secondary mental health services. 25 participants were self-referred in so far as they or a family member initiated contact with the research team. In all cases, acceptance into the study included the agreement of the participant's responsible clinician, who provided clinical and risk information and agreed to be contacted regarding any deterioration in mental state. Attrition was within the prespecified target of 20% for the primary outcome at 12 weeks: 63 (84%) of 75 in the AVATAR group and 61 (81%) of 75 in the supportive counselling group completed assessments (figure 1). At 24 weeks, 57 (76%) participants in the AVATAR group and 58 (77%) in the supportive counselling group completed assessments of primary outcome measures (figure 1).

Unmasking occurred in 28 participants (18·6%), 14 in each study group. When unmasking occurred, subsequent assessments were done by a different researcher. Replacement was not possible in three of these 28 participants (two participants refused to see any other researcher and one participant repeatedly unmasked the replacement researcher).

Overall, there was a greater proportion of men, the mean age was 42·7 years (SD 10·7), and approximately 40% of participants in each group belonged to an ethnic minority population (table 1). Most participants were unemployed and the most common diagnosis was paranoid schizophrenia (table 1). Participants had an average length of illness of 20·1 years (SD 10·8) and all were prescribed antipsychotic medication before the trial, with more than a third prescribed clozapine. There was no difference at baseline or follow-up in the total dose of prescribed antipsychotic medication (olanzapine or chlorpromazine equivalents). The majority of participants (n=117 [78%]) reported hearing multiple voices (mean 3·6 [SD 3·5], range 1 to >20) and most identified the source as of human origin, though only a third said they thought they knew the person responsible.

**Table 1:** Baseline characteristics of the intention-to-treat population

		**Supportive counselling (n=75)**	**AVATAR therapy (n=75)**	**Total (n=150)**
Age (years)		42·9 (11·2)	42·5 (10·1)	42·7 (10·7)
Sex				
	Female	30 (40%)	18 (24%)	48 (32%)
	Male	45 (60%)	57 (76%)	102 (68%)
Ethnicity				
	White British	32 (43%)	26 (35%)	58 (39%)
	Black British	11 (15%)	15 (20%)	26 (17%)
	Black Caribbean	8 (11%)	7 (9%)	15 (10%)
	Black African	6 (8%)	7 (9%)	13 (9%)
	Asian Indian	2 (3%)	2 (3%)	4 (3%)
	Asian Chinese	1 (1%)	0	1 (1%)
	Other	15 (20%)	18 (24%)	33 (22%)
Education				
	Primary	14 (19%)	16 (21%)	30 (20%)
	Secondary or equivalent	30 (40%)	28 (37%)	58 (39%)
	Vocational education	13 (17%)	17 (23%)	30 (20%)
	University degree	18 (24%)	14 (19%)	32 (21%)
Employment				
	Employed (full time or part time)	5 (7%)	7 (9%)	12 (8%)
	Unemployed	64 (85%)	65 (87%)	129 (86%)
	Housewife or husband	1 (1%)	0	1 (1%)
	Student	1 (1%)	3 (4%)	4 (3%)
	Information not provided	2 (3%)	2 (3%)	4 (3%)
Diagnosis				
	Paranoid schizophrenia	58 (77%)	57 (76%)	115 (77%)
	Schizoaffective disorder	8 (11%)	8 (11%)	16 (11%)
	Bipolar disorder	6 (8%)	1 (1%)	7 (5%)
	Unspecific psychosis	3 (4%)	5 (7%)	8 (5%)
	Depression with psychotic symptoms	0	4 (5%)	4 (3%)
Hospital admission				
	Never	8 (11%)	11 (15%)	19 (13%)
	Between one and five times	45 (60%)	43 (57%)	88 (59%)
	More than five times	18 (24%)	19 (25%)	37 (25%)
	Unknown	4 (5%)	2 (3%)	6 (4%)
Length of illness (years)		19·8 (10·8)	20·5 (10·1)	20·1 (10·8)
Olanzapine equivalent of antipsychotic medication (mg/day)		22·5	23·9	23·2
Voice characteristics				
	Number of voices	3·2 (2·9)	3·9 (4·0)	3·6 (3·5)
	Known person	34 (45%)	19 (25%)	53 (35%)
	Personification (human)	62 (83%)	70 (93%)	132 (83%)

Data are mean (SD) or n (%), unless otherwise specified.

Across both therapy groups, 1030 therapy sessions were offered to the participants. The average number of sessions attended was 5·6 (SD 2·8, range 0–10) for AVATAR therapy and 5·1 (3·1, 0–10) for supportive counselling. 103 participants completed a full course of therapy, with a further 27 discontinuing between sessions one and six (figure 1). Reasons for discontinuation varied and included logistical issues (time and distance to travel), physical health problems, or participants reporting that the approach was not relevant or helpful for them (in both groups). There were no cases in either group of therapists discontinuing therapy or treating clinicians requesting discontinuation because of concerns about adverse effects of therapy. 20 participants attended no sessions at all (five in the AVATAR group, 15 in the supportive counselling group). Rates of non-attendance were 23% for supportive counselling and 15% for AVATAR therapy. Fidelity to the manualised approach and skill in delivery was high and did not differ between therapists, with an overall adherence to the manual with a mean score of 18·9 (SD 2·3) out of a maximum of 21 (range 15–21) and average skill rating of 28·2 (SD 1·7) out of a maximum of 30 (range 25–30). The adherence to the supportive counselling manual was high, with an overall mean of 15·2 (SD 1·2) out of a maximum of 16. For the counselling adherence scale in which seven items are scored 0 (no evidence) to 4 (very good evidence), the mean was 21·8 (SD 1·9) out of a maximum of 28, which equates to a rating of greater than 3 (good evidence) across the seven items. Intra-class correlations for inter-rater reliability ranged from 0·78 to 0·98, showing good to excellent agreement

**Figure 2:**
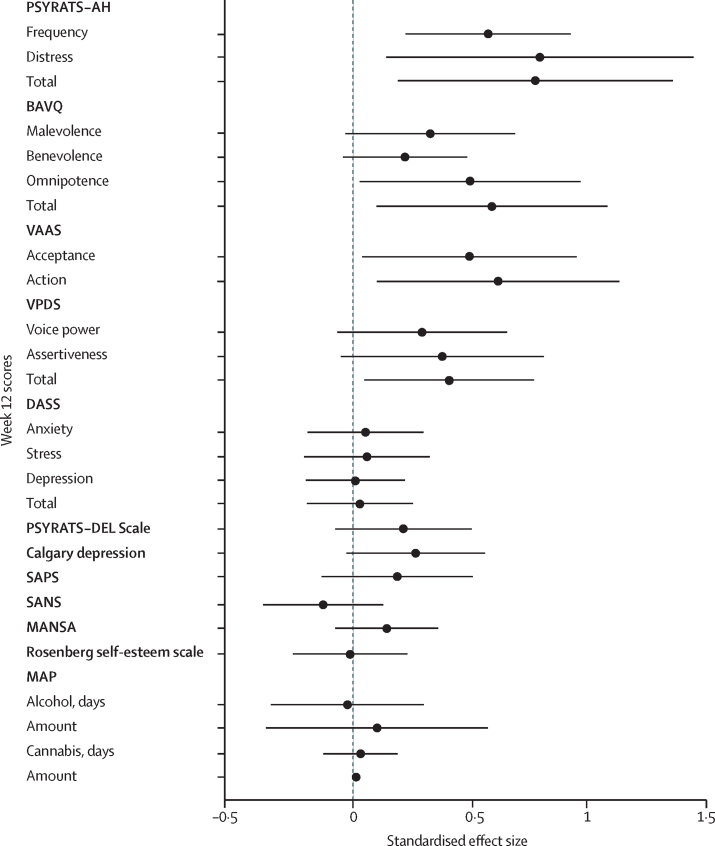
Week 12 effect estimates and 95% CIs Forest plot shows standardised Cohen's *d* effect sizes for AVATAR versus supportive counselling. Positive effect favours AVATAR therapy. All scales reversed, except for VAAS and MANSA. PSYRATS-AH= Psychotic Symptoms Rating Scales–Auditory Hallucinations. BAVQ-R=Beliefs About Voices Questionnaire. VAAS=Voice Acceptance and Action Scale. VPDS=Voice Power Differential Scale. DASS-21=Depression Anxiety and Stress Scale. PSYRATS–DEL=Psychotic Symptoms Rating Scales–Delusions. SAPS=Scale for Assessment of Positive Symptoms. SANS=Scale for Assessment of Negative Symptoms. MANSA=Manchester Quality of Life. MAP=Maudsley Addiction Profile.

**Figure 3:**
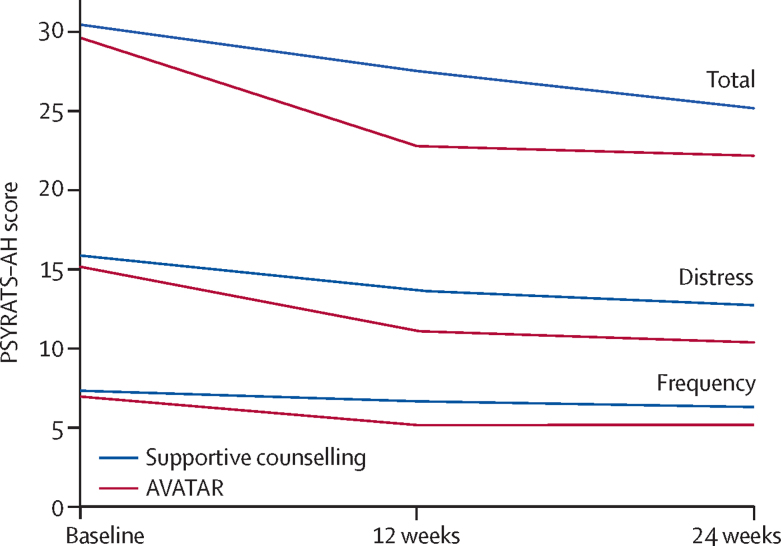
PSYRATS–AH total, distress, and frequency scores

Both the descriptive statistics (table 2) and the effect estimates (figure 2) show that at 12 weeks, AVATAR therapy led to significantly greater reductions in auditory hallucinations than did supportive counselling, as assessed by the PSYRATS–AH total score (estimated mean difference −3·82, SE 1·47, 95% CI −6·70 to −0·94; p=0·009; *d*=0·8). There were also significant differences in reported frequency of voices and reduced distress at 12 weeks (table 2). The trajectories of the PSYRATS–AH measures over time for each group are shown in figure 3.

**Table 2:** Primary and secondary outcomes at baseline, 12 weeks, and 24 weeks

	**Supportive counselling**	**AVATAR therapy**	**Adjusted mean difference (SE)**	**95% CI; p value**
**PSYRATS–AH–Total (0–44)**				
Baseline	30·46 (5·07), n=75	29·63 (4·72), n=75	..	..
12 weeks	27·53 (7·75), n=61	22·79 (10·65), n=63	−3·82 (1·47)	−6·70 to −0·94; p=0·0093
24 weeks	25·18 (10·73), n=58	22·18 (11·12), n=57	−1·55 (1·80)	−5·09 to 1·98; p=0·39
**PSYRATS–AH–Frequency (0–12)**				
Baseline	7·34 (2·07), n=75	6·97 (2·16), n=75	..	..
12 weeks	6·67 (2·28), n=61	5·17 (2·74), n=63	−1·22 (0·38)	−1·97 to −0·48; p=0·0013
24 weeks	6·31 (2·88), n=58	5·18 (3·06), n=57	−0·66 (0·47)	−1·58 to 0·26; p=0·16
**PSYRATS–AH–Distress (0–20)**				
Baseline	15·83 (2·66), n=75	15·17 (3·16), n=75	..	..
12 weeks	13·79 (4·64), n=61	11·10 (5·93), n=63	−2·34 (0·98)	−4·26 to −0·42; p=0·017
24 weeks	12·74 (5·96), n=58	10·39 (6·23), n=57	−1·77 (1·04)	−3·81 to 0·28; p=0·090
**BAVQ-R–Malevolence (0–18)**				
Baseline	12·10 (4·33), n=75	10·77 (4·60), n=75	..	..
12 weeks	10·77 (4·66), n=61	8·34 (5·64), n=63	−1·49 (0·83)	−3·12 to 0·15; p=0·074
24 weeks	8·81 (5·51), n=58	8·09 (5·78), n=57	0·21 (0·93)	−1·61 to 2·04; p=0·82
**BAVQ-R–Benevolence (0–18)**				
Baseline	2·99 (4·30), n=75	3·31 (4·01), n=75	..	..
12 weeks	3·21 (4·55), n=61	2·70 (3·60), n=63	−0·93 (0·56)	−2·03 to 0·17; p=0·10
24 weeks	2·79 (4·12), n=58	2·95 (3·95), n=57	0·069 (0·63)	−1·16 to 1·30; p=0·91
**BAVQ-R–Omnipotence (0–18)**				
Baseline	11·71 (4·10), n=75	10·31 (4·07), n=75	..	..
12 weeks	10·21 (4·31), n=61	7·87 (5·00), n=63	−2·07 (0·99)	−4·01 to −0·12; p=0·038
24 weeks	9·28 (5·37), n=58	7·56 (5·36), n=57	−0·818 (0·85)	−2·47 to 0·84; p=0·33
**BAVQ-R–Total (0–105)**				
Baseline	50·99 (14·07), n=75	46·94 (12·18), n=75	..	..
12 weeks	47·67 (13·70), n=61	39·28 (19·52), n=63	−7·88 (3·34)	−14·43 to −1·33; p=0·018
24 weeks	41·41 (18·03), n=58	36·76 (20·17), n=57	−2·59 (3·26)	−8·98 to 3·80; p=0·43
**VAAS–Acceptance (16–80)**				
Baseline	48·12 (8·44), n=75	50·19 (6·61), n=75	..	..
12 weeks	51·13 (8·75), n=61	55·89 (10·13), n=63	3·80 (1·78)	0·30 to 7·29; p=0·033
24 weeks	52·67 (10·48), n=58	56·31 (11·33), n=57	2·26 (1·89)	−1·44 to 5·97; p=0·23
**VAAS–Action (15–75)**				
Baseline	47·78 (9·76), n=75	49·48 (8·50), n=75	..	..
12 weeks	49·56 (10·03), n=61	54·28 (11·32), n=63	5·69 (2·42)	0·94 to 10·44; p=0·019
24 weeks	51·76 (10·85), n=58	55·05 (12·09), n=57	1·94 (1·68)	−1·36 to 5·23; p=0·25
**VPDS–Voice power (1–5)**				
Baseline	3·03 (1·38), n=60	3·09 (1·29), n=53	..	..
12 weeks	2·97 (1·50), n=58	2·61 (1·41), n=57	−0·39 (0·25)	−0·88 to 0·09; p=0·11
24 weeks	2·76 (1·50), n=55	2·67 (1·39), n=55	−0·42 (0·27)	−0·94 to 0·11; p=0·12
**VPDS–Assertiveness (1–5)**				
Baseline	3·28 (1·37), n=60	3·13 (1·52), n=53	..	..
12 weeks	3·31 (1·40), n=58	2·54 (1·38), n=57	−0·55 (0·32)	−1·17 to 0·07; p=0·084
24 weeks	3·05 (1·35), n=55	2·55 (1·33), n=55	−0·26 (0·28)	−0·81 to 0·29; p=0·35
**VPDS–Total (7–35)**				
Baseline	22·37 (7·30), n=60	22·13 (6·64), n=53	..	..
12 weeks	21·21 (7·40), n=58	18·30 (7·93), n=57	−2·86 (1·29)	−5·39 to −0·34; p=0·026
24 weeks	20·24 (7·75), n=55	17·95 (7·82), n=55	−2·45 (1·47)	−5·33 to 0·43; p=0·09
**PSYRATS–DEL (0–24)**				
Baseline	13·83 (6·19), n=72	11·91 (6·52), n=75	..	..
12 weeks	12·85 (5·13), n=60	10·33 (7·14), n=63	−1·38 (0·95)	−3·25 to 0·48; p=0·15
24 weeks	10·98 (6·96), n=58	9·29 (7·29), n=55	0·24 (1·16)	−2·04 to 2·51; p=0·84
**Calgary depression scale (0–27)**				
Baseline	8·08 (5·45), n=73	7·84 (5·64), n=74	..	..
12 weeks	7·41 (4·82), n=61	5·43 (5·20), n=63	−1·48 (0·84)	−3·12 to 0·16; p=0·076
24 weeks	6·67 (5·01), n=58	5·96 (5·28), n=57	−0·34 (0·86)	−2·02 to 1·35; p=0·69
**DASS-21–Anxiety (0–21)**				
Baseline	8·71 (5·47), n=72	7·53 (5·27), n=73	..	..
12 weeks	6·86 (5·53), n=61	5·60 (4·58), n=62	−0·29 (0·68)	−1·62 to 1·04; p=0·67
24 weeks	6·31 (4·58), n=58	6·03 (5·18), n=57	0·31 (0·78)	−1·22 to 1·83; p=0·69
**DASS-21–Stress (0–21)**				
Baseline	8·82 (4·70), n=72	8·95 (5·87), n=73	..	..
12 weeks	7·77 (5·52), n=61	7·08 (5·02), n=62	−0·32 (0·72)	−1·74 to 1·10; p=0·66
24 weeks	7·64 (4·89), n=58	6·65 (5·11), n=57	−1·04 (0·75)	−2·50 to 0·43; p=0·17
**DASS-21–Depression (0–21)**				
Baseline	9·53 (6·16), n=72	8·75 (6·62), n=73	..	..
12 weeks	8·00 (5·82), n=61	6·79 (5·91), n=62	−0·066 (0·69)	−1·41 to 1·28; p=0·92
24 weeks	6·95 (6·01), n=58	7·08 (5·47), n=57	0·75 (0·84)	−0·91 to 2·40; p=0·38
**DASS-21–Total (0–63)**				
Baseline	27·06 (14·64), n=72	25·22 (15·98), n=73	..	..
12 weeks	22·63 (15·52), n=61	19·46 (14·08), n=62	−0·45 (1·77)	−3·91 to 3·02; p=0·80
24 weeks	20·90 (13·81), n=58	19·76 (14·70), n=57	0·12 (2·08)	−3·95 to 4·20; p=0·95
**SAPS (0–170)**				
Baseline	42·18 (18·47), n=73	37·43 (16·46), n=75	..	..
12 weeks	39·93 (20·75), n=61	32·10 (20·50), n=62	−3·32 (2·89)	−8·99 to 2·35; p=0·25
24 weeks	34·66 (19·49), n=58	32·09 (21·47), n=57	2·32 (3·04)	−3·63 to 8·28; p=0·44
**SANS (0–125)**				
Baseline	29·19 (19·89), n=75	28·21 (17·71), n=75	..	..
12 weeks	25·61 (17·66), n=61	26·67 (19·79), n=63	2·39 (2·45)	−2·42 to 7·20; p=0·33
24 weeks	25·83 (17·62), n=58	25·83 (19·16), n=57	1·90 (2·55)	−3·10 to 6·90; p=0·46
**MANSA (16–112)**				
Baseline	51·49 (12·15), n=70	51·16 (12·87), n=72	..	..
12 weeks	52·95 (11·69), n=59	55·64 (11·62), n=61	1·80 (1·40)	−0·94 to 4·54; p=0·19
24 weeks	53·03 (13·11), n=55	54·20 (11·85), n=57	0·36 (1·58)	−2·74 to 3·46; p=0·82
**Rosenberg self-esteem (10–40)**				
Baseline	25·65 (6·35), n=64	25·12 (6·36), n=65	..	..
12 weeks	26·95 (5·72), n=60	27·50 (6·09), n=61	0·08 (0·79)	−1·47 to 1·63; p=0·92
24 weeks	27·60 (6·36), n=57	27·02 (6·55), n=56	−0·76 (0·99)	−2·70 to 1·17; p=0·44
**MAP: alcohol days used (past 30 days)**				
Baseline	3·32 (6·55), n=71	1·57 (3·76), n=75	..	..
12 weeks	3·16 (6·63), n=61	2·37 (5·41), n=62	0·13 (0·89)	−1·62 to 1·87; p=0·89
24 weeks	2·51 (5·21), n=57	1·84 (4·08), n=57	0·49 (0·70)	−0·89 to 1·87; p=0·48
**MAP: alcohol units consumed (past 30 days)**				
Baseline	16·18 (38·95), n=71	10·14 (27·77), n=75	..	..
12 weeks	20·70 (57·22), n=61	14·76 (38·09), n=62	−3·45 (8·14)	−19·40 to 12·51; p=0·67
24 weeks	14·19 (31·34), n=57	26·31 (123·19), n=57	15·65 (16·56)	−16·80 to 48·11; p=0·35
**MAP: cannabis days used (past 30 days)**				
Baseline	1·63 (6·55), n=70	1·93 (6·60), n=75	..	..
12 weeks	1·43 (5·74), n=61	1·37 (5·72), n=62	−0·21 (0·53)	−1·25 to 0·83; p=0·69
24 weeks	1·02 (4·54), n=57	1·63 (5·88), n=57	0·36 (0·79)	−1·18 to 1·91; p=0·65
**MAP: cannabis joints smoked (past 30 days)**				
Baseline	3·01 (13·86), n=69	7·33 (38·40), n=73	..	..
12 weeks*	2·92 (12·87), n=61	8·26 (41·49), n=62	−0·38 (0·28)	−1·03 to 0·28; p=0·22
24 weeks	1·98 (9·09), n=57	7·77 (34·68), n=57	1·81 (2·43)	−2·95 to 6·57; p=0·46

Data are mean score (SD), number of observations. Numbers of observations differ from the maximum when participants did not complete the measure. PSYRATS–AH=Psychotic Symptoms Rating Scale–Auditory Hallucinations. BAVQ-R=Beliefs About Voices Questionnaire. VAAS=Voice Acceptance and Action Scale. VPDS=Voice Power Differential Scale. PSYRATS–DEL=Psychotic Symptoms Rating Scale–Delusions. DASS-21=Depression Anxiety and Stress Scale. SAPS=Scale for Assessment of Positive Symptoms. SANS=Scale for Assessment of Negative Symptoms. MANSA=Manchester Short Assessment of Quality of Life. MAP=Maudsley Addiction Profile.

* Mixed model for week 12 cannabis use (amount) as defined in SAP did not converge, and results come from linear regression with no random effects using SE estimates that allow for clustering defined by therapist.

Nine participants reported a complete absence of voices during the preceding week at the week 12 assessment (seven in the AVATAR therapy group and 2 in the supportive counselling group), and 14 participants reported an absence at 24 weeks (eight in the AVATAR group and six in the supportive counselling group).

At 12 weeks, there were significant differences in reductions in the secondary outcomes of perceived omnipotence of voices, as measured by BAVQ-R–Omnipotence, VAAS–Acceptance, and VAAS–Action (table 2).

At 24 weeks follow-up, the improvements in the scores on PSYRATS-AH, BAVQ, and VAAS in the AVATAR group were maintained (table 2; figure 3). The supportive counselling group, however, continued to improve such that there were no significant differences between the two groups by this point. No significant differences between the two groups were observed for any of the other secondary outcomes at either 12 or 24 weeks.

During 24 weeks, five participants in the AVATAR therapy group and seven in the supportive counselling were admitted to hospital, and one additional participant in each group required acute home treatment. Severe mental or physical health deterioration was observed in three participants (one in the AVATAR group and two in the supportive counselling group). Violent incidents were reported in the clinical records of five participants (three in the AVATAR therapy group and two in the supportive counselling group), and in one of these the participant was the victim. There were no recorded incidents of self-harm or suicide attempts. The independent data monitoring and ethics committee found none of the adverse events to be attributable to AVATAR therapy or supportive counselling.

## Discussion

AVATAR therapy was feasible to deliver, acceptable to participants, and did not result in any adverse events that could be attributed to the therapy. The study corroborated the primary hypothesis concerning clinical efficacy by showing a rapid and sustained reduction in the severity of auditory verbal hallucinations by end of therapy at week 12 that was significantly superior to that achieved by supportive counselling. The observed outcomes at week 12 were larger than expected, with a between-group effect size for the primary outcome of 0·8. This is a larger effect than the mean effect reported for voices by the most relevant recent meta-analysis of cognitive behavioural therapy for psychosis (d=0·46),^2^ which is very likely to be an upper estimate given that it includes data from the earlier AVATAR pilot study,^10^ and given that few trials of other psychological therapies for psychosis have included an active control condition. Our second and third hypotheses were also largely supported, in that AVATAR therapy had a positive and significant on omnipotence, and that these positive effects on voices were sustained at 24 weeks. However, it had no significant effect on the reported malevolence of voices.

It is important to note that the trial involves a sample of people suffering from persistent psychoses who reported unremitting and very distressing auditory hallucinations for at least the previous 12 months, despite regular supervision and continuing pharmacological treatment. More than a third of all patients across both therapy groups had a clinical record of treatment resistance and were prescribed clozapine before the start of the study. The average number of voices in people with psychosis ranges between 3·2 and 4·3 when uncountable numbers are excluded,^27^ a figure that is consistent with the average noted in our sample. In testing the therapy as an intervention that would have wide applications, we decided not to pre-specify the number of voices, but instead to ask participants to select the voice that they most wished to influence, with our clinical impression being that positive changes can generalise from the target voice.

In terms of limitations, the absence of a treatment-as-usual control condition complicates interpretation of the absence of a significant difference between the two groups at 24 weeks. Although, as hypothesised, the large effect of AVATAR is maintained after therapy up to 24 weeks, participants who received supportive counselling show a small improvement after therapy, reducing the between-group difference. There are two main possibilities to be considered. First, that the results reflect regression to the mean in both groups. We consider this to be unlikely, in that the participants were selected for persistent symptoms and were not recruited in crisis, thus the baseline state should be relatively stable without an intervention. This possibility would also not explain the more rapid improvement in the AVATAR therapy group. Of note in our study is the finding that, in both groups, improvements on the PSYRATS score at week 24 follow-up were well in excess of the five-point change defined, a priori, as a clinically significant reduction,^11^ with a larger numerical change in the AVATAR therapy group than in the supportive counselling group.

However, although it was less rapidly effective, supportive counselling as delivered in this trial could probably also be beneficial for treatment of auditory hallucinations. Supportive counselling is a control condition with non-specific factors that, compared with no treatment, can be effective in its own right.^28^ There is evidence for a small positive effect for supportive counselling and befriending in psychosis, by contrast with smaller or zero effects of treatment as usual.^29^ When designing the supportive counselling intervention, we made every effort to ensure that this therapy was delivered competently, with close weekly supervision provided by the trial therapy coordinator, which included regular use of live recordings. Furthermore, supportive counselling in this trial went beyond a simple attentional control and addressed practical concerns about living with psychosis, finding ways to improve current quality of life, coming to terms with past trauma, and identifying personal resources and qualities. The scores of counselling competency delivered in the control group suggest that this therapy was delivered proficiently. Provision of audiotaped recordings of the best 10 min of each supportive counselling session and encouragement of participants to reflect on these recordings as an attempt to control for the homework element of AVATAR therapy went beyond what is usually provided in a therapist attention control, which is why we consider this to be an augmented form of supportive counselling. Whether comparisons of AVATAR and supportive counselling with treatment as usual would favour AVATAR cannot be addressed with this study. The issue of when best to compare new treatments with treatment as usual is discussed by Gold and colleagues,^28^ who come to the broad conclusion that this approach is most appropriate at early phase 2, and much larger, subsequent pragmatic trials with active controls are useful at an intermediate stage along this pathway. We originally considered a three-arm study but rejected it on the grounds of the resources required and the stage of AVATAR research. In future studies, different options for design and control conditions, including novel designs and how and whether to include a treatment-as-usual condition, should be carefully considered.^28^

Another limitation is the fact that the study was done in only one centre by skilled therapists with substantial expertise in the psychological treatment of psychosis, which limits generalisation to other centres or to delivery by a wider mental health workforce. At this stage, we also cannot be certain that the outcomes of AVATAR therapy would be superior to an equivalent relational therapy that did not have the added costs of audio-visual technology.

Given that AVATAR therapy was effective at the end of therapy but had less comparative benefit at follow-up, could the immediate effect of AVATAR therapy be enhanced by any changes in the therapy approach? As delivered, and following the original AVATAR pilot study, AVATAR therapy was a very brief and tightly focussed intervention. Longer-term benefits might require additional sessions or effectiveness might be increased by a higher dose (ie, a more intensive focus over longer than 3–6 sessions) on potential effective mechanisms, such as increasing control and reducing perceived omnipotence. Even with a brief intervention such as AVATAR therapy, it would also be relevant to examine in future research the contribution of the different intervention components (eg, exposure and anxiety reduction, assertiveness and control, self-esteem, and trauma re-processing) to the reduction in auditory hallucinations.

How do the effects of AVATAR therapy compare with other therapies for auditory verbal hallucinations? To our knowledge, the AVATAR therapy effects on frequency and severity of auditory verbal hallucinations, as assessed by PSYRATS–AH, are stronger than more general cognitive behavioural therapy for psychosis, as applied to voices.^2^ The COMMAND trial,^7^ one of the most successful trials for auditory verbal hallucinations to date, employed a more targeted cognitive behavioural therapy approach for command hallucinations, and showed sustained benefits for compliance with commands (the primary treatment target), although not on frequency or distress associated with the hallucinations (PSYRATS–AH) or omnipotence as measured by the BAVQ-R. However, the COMMAND trial involved a much longer-term therapy, delivered over 25 sessions and 9 months, with a different treatment goal and participant selection criteria, and where the comparator was treatment as usual rather than an active treatment control. A recently published pilot study of Relating Therapy^1^ has also reported encouraging preliminary results on reducing voice-related distress when compared with treatment as usual.^1^ Like AVATAR therapy, Relating Therapy focuses on dialogue with voices and increasing assertiveness, albeit without a digital avatar, but differs in being of longer duration and with a stronger focus on wider social relationships.^1^

AVATAR therapy is a brief therapy for persistent, distressing voices that makes creative and novel use of digital representations of psychotic experiences to provide a controlled but realistic therapeutic encounter, enabling dialogue and change. In a rapid development, from an initial pilot to the first powered randomised controlled trial, AVATAR therapy has shown large, clinically worthwhile benefits for voice hearers. In future reports, we will examine secondary hypotheses concerning mediation and moderation of effects, heath economic analysis, the participant experience of the virtual reality aspects of the avatar (such as sense of presence), and processes of therapy delivery.

**This online publication has been corrected. The corrected version first appeared at thelancet.com/psychiatry on November 29, 2017**

## References

[1] Hayward M, Jones AM, Bogen-Johnston L, Thomas N, Strauss C (2017). Relating Therapy for distressing auditory hallucinations: a pilot randomized controlled trial. Schizophr Res.

[2] van der Gaag M, Valmaggia LR, Smit F (2014). The effects of individually tailored formulation-based cognitive behavioural therapy in auditory hallucinations and delusions: a meta-analysis. Schizophr Res.

[3] Waters F, Allen P, Aleman A (2012). Auditory hallucinations in schizophrenia and nonschizophrenia populations: a review and integrated model of cognitive mechanisms. Schizophr Bull.

[4] Aleman A, Laroi F (2011). Insights into hallucinations in schizophrenia: novel treatment approaches. Expert Rev Neurother.

[5] Haddock G, Eisner E, Boone C, Davies G, Coogan C, Barrowclough C (2014). An investigation of the implementation of NICE-recommended CBT interventions for people with schizophrenia. J Ment Health.

[6] Chadwick P, Birchwood M (1994). The omnipotence of voices—a cognitive approach to auditory hallucinations. Brit J Psychiat.

[7] Birchwood M, Michail M, Meaden A (2014). Cognitive behaviour therapy to prevent harmful compliance with command hallucinations (COMMAND): a randomised controlled trial. Lancet Psychiatry.

[8] Paulik G (2012). The role of social schema in the experience of auditory hallucinations: a systematic review and a proposal for the inclusion of social schema in a cognitive behavioural model of voice hearing. Clin Psychol Psychother.

[9] Longden E, Corstens D, Escher S, Romme M (2012). Voice hearing in a biographical context: a model for formulating the relationship between voices and life history. Psychosis.

[10] Leff J, Williams G, Huckvale MA, Arbuthnot M, Leff AP (2013). Computer-assisted therapy for medication-resistant auditory hallucinations: proof-of-concept study. Br J Psychiatry.

[11] Craig TK, Rus-Calafell M, Ward T (2015). The effects of an Audio Visual Assisted Therapy Aid for Refractory auditory hallucinations (AVATAR therapy): study protocol for a randomised controlled trial. Trials.

[12] Craig T, Ward T, Rus-Calafell M, Pradhan B, Pinninti N, Rathod S (2016). AVATAR therapy for refractory auditory hallucinations. Brief interventions for psychosis: a clinical compendium.

[13] Lewis S, Tarrier N, Haddock G (2002). Randomised controlled trial of cognitive-behavioural therapy in early schizophrenia: acute-phase outcomes. Br J Psychiatry Suppl.

[14] Rogers CR (1957). The necessary and sufficient conditions of therapeutic personality change. J Consult Psychol.

[15] Hill A (2011). Curriculum for counselling for depression. http://webarchive.nationalarchives.gov.uk/20160302160209.

[16] Haddock G, McCarron J, Tarrier N, Faragher EB (1999). Scales to measure dimensions of hallucinations and delusions: the psychotic symptom rating scales (PSYRATS). Psychol Med.

[17] Woodward TS, Jung K, Hwang H (2014). Symptom dimensions of the psychotic symptom rating scales in psychosis: a multisite study. Schizophr Bull.

[18] Chadwick P, Lees S, Birchwood M (2000). The revised Beliefs About Voices Questionnaire (BAVQ-R). Brit J Psychiat.

[19] Shawyer F, Ratcliff K, Mackinnon A, Farhall J, Hayes SC, Copolov D (2007). The voices acceptance and Action Scale (VAAS): pilot data. J Clin Psychol.

[20] Birchwood M, Meaden A, Trower P, Gilbert P, Plaistow J (2000). The power and omnipotence of voices: subordination and entrapment by voices and significant others. Psychol Med.

[21] Andreasen NC (1984). The scale for the assessment of positive symptoms (SAPS).

[22] Lovibond PF, Lovibond SH (1995). The structure of negative emotional states—comparison of the Depression Anxiety Stress Scales (DASS) with the Beck Depression and Anxiety Inventories. Behav Res Ther.

[23] Addington D, Addington J, Matickatyndale E (1993). Assessing depression in schizophrenia—the Calgary Depression Scale. Brit J Psychiat.

[24] Rosenberg M (1965). Society and the adolescent self-image.

[25] Priebe S, Huxley P, Knight S, Evans S (1999). Application and results of the Manchester Short Assessment of Quality of Life (MANSA). Int J Soc Psychiatr.

[26] Marsden J, Gossop M, Stewart D (1998). The Maudsley Addiction Profile (MAP): a brief instrument for assessing treatment outcome. Addiction.

[27] McCarthy-Jones S, Trauer T, Mackinnon A, Sims E, Thomas N, Copolov DL (2012). A new phenomenological survey of auditory hallucinations: evidence for subtypes and implications for theory and practice. Schizophr Bull.

[28] Gold SM, Enck P, Hasselmann H (2017). Control conditions for randomised trials of behavioural interventions in psychiatry: a decision framework. Lancet Psychiatry.

[29] Turner DT, van der Gaag M, Karyotaki E, Cuijpers P (2014). Psychological interventions for psychosis: a meta-analysis of comparative outcome studies. Am J Psychiat.

