# Corrections

**DOI:** 10.1016/S2215-0366(17)30473-X

**Published:** 2018-01

C*raig TKJ, Rus-Calafell M, Ward T, et al. AVATAR therapy for auditory verbal hallucinations in people with psychosis: a single-blind, randomised controlled trial.* Lancet Psychiatry *2017; **5:** 31–40*—The Summary has been updated to include “(invented by Julian Leff in 2008)”. The Acknowledgments have been updated to include additional support: “PAG and TKJC acknowledge support for partial funding of sessions and for infrastructure support from the National Institute for Health Research (NIHR) Biomedical Research Centre at South London and Maudsley NHS Foundation Trust and King's College London. The views expressed are those of the author(s) and not necessarily those of the NHS, the NIHR or the Department of Health.” These corrections have been made to the online version as of Nov 29, 2017.

